# *Mesobuthus eupeus* venom modulates colorectal carcinoma signaling pathways and induces apoptosis

**DOI:** 10.1007/s12032-025-02689-2

**Published:** 2025-04-14

**Authors:** Havva Nur Canak, Kemal Bas, Ersen Aydın Yağmur, Serdar Karakurt

**Affiliations:** 1 Faculty of Science, Department of Biochemistry, Selcuk University, Konya, Türkiye; 2 Department of Plant and Animal Production, Alasehir Vocational High School, Manisa Celal Bayar University, Manisa, Türkiye

**Keywords:** Colorectal cancer, *Mesobuthus eupeus*, Venom, Cytotoxicity, Molecular mechanism

## Abstract

Colorectal cancer (CRC) is a significant global health concern, often challenging to treat effectively with conventional methods and burdened by adverse effects. Scorpion venoms offer a unique avenue for exploration, given their ability to disrupt the cell cycle, inhibit growth, and trigger apoptosis. This study delves into the impact of *Mesobuthus eupeus* (*M. eupeus*) scorpion venom on the proliferation and progression of colorectal cancer at the molecular level. The total protein concentration in the venom (607.5 µg/mL) also emphasized the rich composition and potential for therapeutic applications. The study reveals that *M. eupeus* venom effectively reduced the proliferation of DLD-1 and HT-29 colorectal cancer cells in a dose-dependent manner with IC_50_ values of 4.32 and 7.61 µg/mL, respectively. The venom also impedes cell migration, diminishes colony formation, and triggers apoptosis in the cancer cells. The venom also induced early and late apoptosis in the two cancer cell lines. The human colorectal cancer and apoptotic pathways were clarified at the molecular level using pathway panels, which revealed that 16 genes involved in colorectal cancer increased while 23 decreased. In the HT-29 cell line, 57 genes increased, and 1 decreased following venom treatment. Besides, the mRNA expression of 19 genes involved in the apoptotic pathway was increased, while 22 were reduced in DLD-1 cells. This study underscores the potential of *M. eupeus* venom as a natural therapeutic approach in the quest for cancer treatments.

**Supplementary Information:**

The online version contains supplementary material available at 10.1007/s12032-025-02689-2.

## Introduction

Cancer, characterized by uncontrolled cell proliferation, metastasis, and invasion, stands as a significant global health challenge [1]. Among leading causes of mortality, it ranks second only to cardiovascular diseases [2, 3]. Colon cancer, a prevalent malignancy, is prominent following lung and prostate cancers in men and breast cancer in women [4]. Colorectal cancer is considered one of the most common cancers worldwide. According to the World Cancer Research Fund's statistics from 2020, colorectal cancer was the third most common cancer globally, with an estimated 1.9 million new cases reported [5].

The intricate progression of colon cancer involves genetic alterations, from early benign polyps triggered by diminished *APC* gene expression to the formation of aggressive malignant tumors driven by mutations in key genes such as *KRAS*, *SMAD4*, and *TP53* [6]. While standard treatments like surgery, radiotherapy, chemotherapy, and immunotherapy are effective, they present challenges, including resistance development and collateral damage to healthy cells [7]. The quest for alternative methods in colon cancer treatment stems from the limitations of conventional therapies, including side effects, resistance development, and impact on patient’s well-being [8]. Natural compounds and novel approaches hold promise in addressing these challenges, offering the potential for enhanced efficacy, reduced side effects, and a more holistic patient-centered approach. Embracing alternative methods can broaden treatment options, improve outcomes, and prioritize the overall quality of care in colon cancer management [9]. Thus, exploring alternative therapeutic avenues, such as natural sources like plants and animal venoms, gains significance. Exploring novel therapeutic approaches has become a hallmark in combating complex diseases like cancer. Among the diverse sources of potential therapeutic agents, scorpion venoms have emerged as intriguing candidates due to their unique bioactive components. Scorpion venoms, renowned for their potent and selective peptides, enzymes, and low molecular weight molecules, have garnered increasing attention for their potential efficacy in various medical applications [7]. Three different methods have been used to extract venom from the scorpions, including maceration, manual, and electrical stimulation. The maceration method is the extraction process of the powder obtained by grinding the scorpion telson with physiological saline (FTS) at + 4 °C for 72 h [10]. The manual method stimulates the abdomen to release scorpion venom [11]. The method of milking with electrical current is obtaining the venom involuntarily from the scorpion with an electric current given to the telson region of live scorpions with an electric source of 0–50 V [12].

**Fig. 1:**
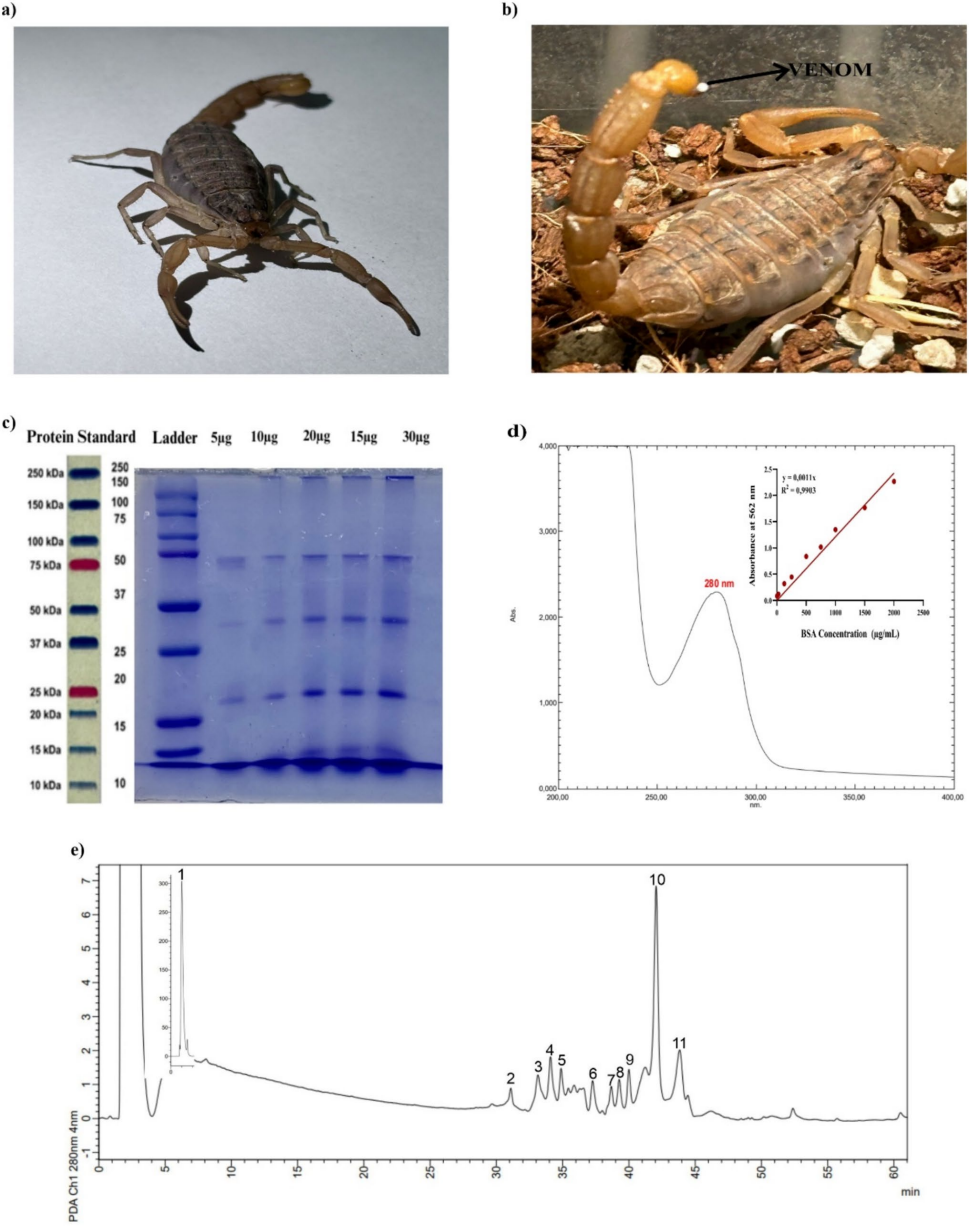
**a** The appearance of *M. eupeus* species scorpion. **b** The sting and venom at the tip of the stinger of *M. eupeus*. **c** The electrophoretic profile of venoms was analyzed on polyacrylamide gel in the presence of SDS under reducing conditions. Lane 1: molecular mass markers, Lane 2–6 different concentrations of venom ranging 5–30 µg. **d** UV–visible spectrum of *M. eupeus* venom and analysis and of protein quantity of *M. eupeus* venom using the BCA method. **e** Separation by HPLC of *M. eupeus* venom. The soluble portion of scorpion venom (1 mg of protein) was subjected to chromatographic separation using a reverse-phase C-18 column. The separation was achieved by applying a linear gradient of solvent A (0.1% trifluoroacetic acid in dH_2_O) to solvent B (0.10% TFA in acetonitrile), reaching 60% solvent B over a 60-min period

The intricate composition of scorpion venoms, shaped by evolution's selective forces, presents an array of compounds with remarkable biological properties [13]. Among these intriguing sources, the venom of *M. eupeus*, a scorpion species prevalent in regions including Syria, Iran, and Southeastern Anatolia in Turkey, has garnered attention for its potential efficacy in diverse medical applications. Carapace and mesosoma reddish brown and black pigmented with dark spots and longitudinal stripes present; metasoma, telson, pedipalps, and legs reddish brown, only dorsal of metasomal segment V fuscous; ventral carinae of metasomal dark colored of this scorpion species [14]. Their length averages around 3.9–6.0 cm. The pectine organ consists of 18–21 teeth in females and 21–25 teeth in males [15, 16]. Genital operculum in females is generally not as long and wide, and tibial spurs on legs III and IV are moderate, with three straight and parallel carinae existing on tergites III–IV in this species **(**Fig. 1a**)**. This species is distributed in Armenia, Azerbaijan, Georgia, Iran (West Azerbaijan Province), Russia, and Turkey [14]. It is recorded from Ağrı, Artvin, Erzurum, Iğdır, and Van provinces in Turkey [15].

This study aims to determine the potential use of scorpion venom in treating human colorectal cancer by assessing cell viability, metastasis, and colony formation potential in human colorectal cancer cells and healthy colon epithelial cells under in vitro conditions. It also aims to elucidate the molecular mechanisms underlying cell death and colorectal cancer development.

## Results and discussion

The scorpions of *M. eupeus* species **(**Fig. 1a**)** were nocturnally collected. Venom extraction involved anesthetizing scorpions with carbon dioxide, followed by electrical stimulation (12 V, 25 amperes) of their telsons **(**Fig. 1b**).** Collected venom was diluted in 0.85% NaCl physiological saline, lyophilized, and stored at − 20 °C for subsequent experimental procedures.

The electrophoretic profile of *M. eupeus* venoms was analyzed by SDS-PAGE electrophoresis [18], confirming the presence of proteins/peptides with distinct molecular weights ranging from 5 to 50 kDa in the venom composition **(**Fig. 1c**)**. A specific maximum peak of the venom was observed at 280 nm with a UV–visible spectrophotometer **(**Fig. 1d**)**. The quantification of total proteins in the venom was calculated as 607.5 µg/mL, referencing a BCA [17] standard curve **(**Fig. 1d**)**. Subsequently, HPLC analysis was performed at 280 nm based on this observation. The HPLC analysis revealed the presence of 11 different proteins/peptides in various fractions and time points within the venom **(**Fig. 1e**)**.

Colorectal cancer (CRC) stands out as a highly lethal malignancy for both genders, prompting substantial mortality annually [17, 18]. The efficacy of conventional therapeutic approaches for this cancer type remains insufficient, coupled with their frequent occurrence of adverse effects. This limitation has spurred the exploration of alternative strategies to mitigate these side effects and develop more effective treatment options [19]. Among these avenues, investigations centered around scorpion venoms, notably those rich in diverse bioactive compounds such as peptides and proteins, have garnered attention for their potential therapeutic applications [20]. The toxicity of venom can vary from region to region and even within the same species [10]. Factors such as the transport conditions of telsons used as venom sources, drying method, storage, and usage duration can also influence venom toxicity [12]. In a study by Oukkache et al. [21], characterization and lethal dose (LD50) were conducted on venoms obtained through manual and electrical stimulation methods for collecting scorpion venoms. It was found that venom extracted manually contained unwanted hemolymph-derived substances, and the venom obtained through electrical stimulation had a threefold higher LD50 value compared to manually collected venom. Characterization studies revealed that manually collected venom contained an additional 75 kDa band not present in the electrophoretic profile of electrically extracted venom. The absorption profiles at 280 nm showed that manually collected venoms had two additional absorption peaks (220–380 and 520–600 nm) not found in venoms extracted via electrical stimulation. This study demonstrated characteristic and lethal dose differences between venom extracted by manual stimulation and electrical stimulation, with the latter providing more accurate results [21]. Research is being conducted on using scorpion venom proteins/peptides in cancer treatment. Some proteins or peptides isolated from venoms specifically bind to the cancer cell membrane, affecting cancer cell migration and proliferation [22]. Maleki and colleagues conducted characterization experiments for isolating toxic peptides from the venom of Hemiscorpius lepturus, an Iranian scorpion, using gel filtration, ion exchange, and reverse-phase high-performance liquid chromatography (RP-HPLC). They identified seven bands with molecular weights between 10 and 100 kDa and a band smaller than 10 kDa from crude venom. In addition, two peaks, HL2153 and HL2155, indicative of high-purity and single-band profiles, were detected after RP-HPLC [23]. In this study, 11 different proteins/peptides from the venom of *M. eupeus* were analyzed using HPLC. The differences are attributed to the scorpion species and the methods used. Khoobdel et al. analyzed the proteins in *M. eupeus* venom using SDS-PAGE. They identified 12 bands ranging from 5 to 140 kDa using a 15% polyacrylamide gel [24]. The protein bands were detected to be between 50, 25–37, and 15–20 kDa, but no specific differences were observed below 15 kDa. These differences are due to gel percentages and the markers used.

**Fig. 2:**
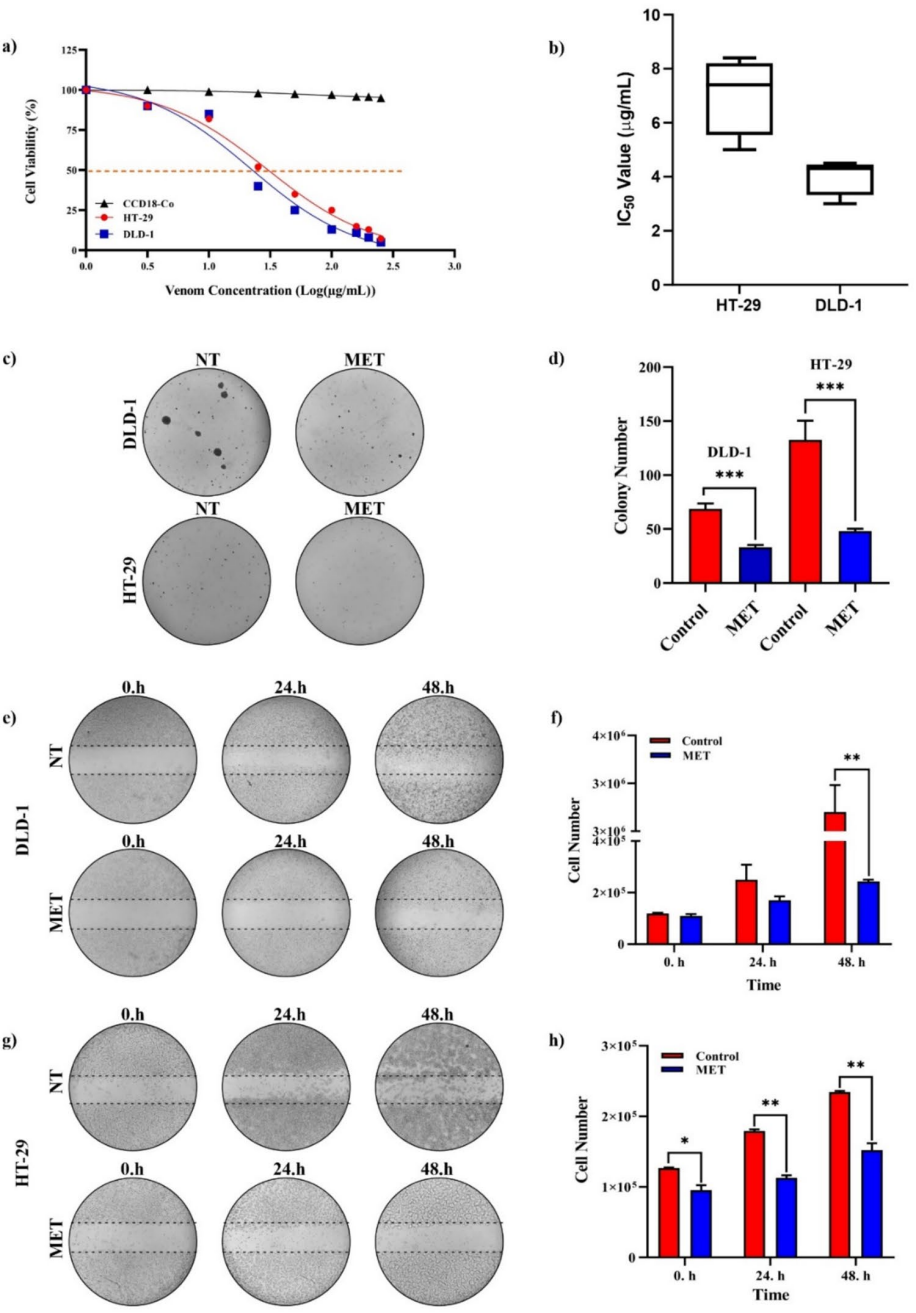
Cytotoxicity, colony formation, and wound healing effects of *M. eupeus* venom on cell lines. **a** Sigmoidal graph of cell viability against log concentration of scorpion venom. **b** IC_50_ values (µg/mL) of HT-29 and DLD-1 cell lines. **c** Colony images of DLD-1 and HT-29 cell lines. **d** Graph of colony count comparing *M. eupeus* venom with the control group. **e** and **g** Images of in vitro wound healing experiment at 0, 24, and 48 h. **f**, **h** Graph of cell count at 0, 24, and 48 h comparing *M. eupeus* venom with the control group. Results are presented as the mean ± SDV of three independent experiments. *p < 0.01, **p < 0.001, and ***p < 0.0001

**Fig. 3:**
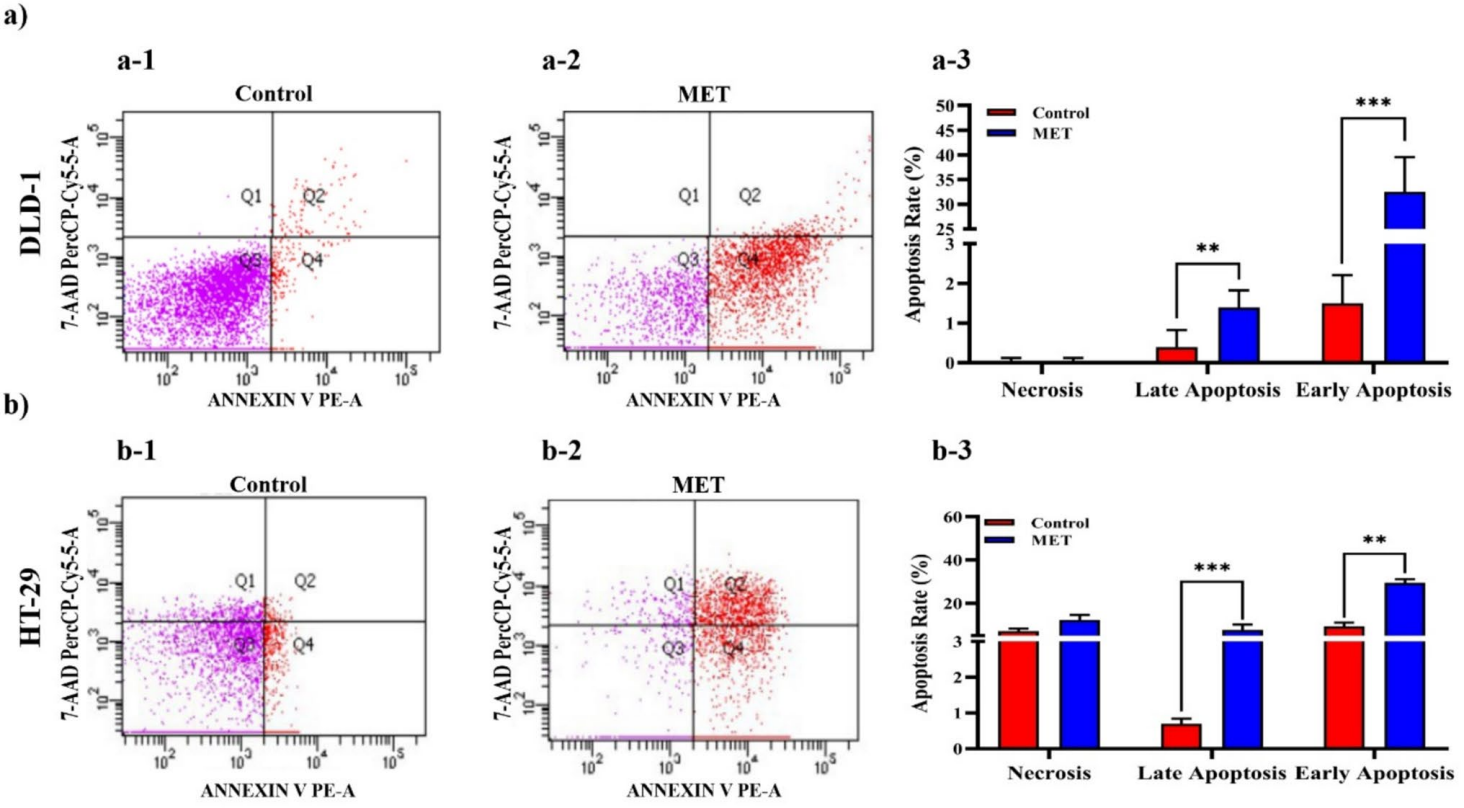
Effects of *M. eupeus* venom on apoptosis in DLD-1 and HT-29 cell lines. **a-1** and **b-1** Flow cytometry images of cells in the control group (Non-treated). **a-2** and **b-2** Flow cytometry images of cells treated with *M. eupeus* venom (Q1: necrotic cells; Q2: late apoptotic cells; Q3: live cells; Q4: early apoptotic cells). **a-3** and **b-3** Ratio of necrosis, early apoptosis, and late apoptosis compared to the control group for *M. eupeus* venom. Results are presented as the mean ± SDV of three independent experiments. **p < 0.001, and ***p < 0.0001

The *M. eupeus* venom exhibited a dose-dependent inhibition of cell viability in hCRC cells, DLD-1 and HT-29, with calculated IC_50_ values of 4.32 and 7.61 µg/mL (Fig. 2a, b), respectively. However, there was no observed cytotoxic effect on the healthy human colon epithelial cells CCD18-Co (IC_50_ > 250 µg/mL) at these concentrations **(**Fig. 2a**)**. Gerges et al. determined the IC_50_ value of Smp43 scorpion peptide as 4.11 µg/mL for colorectal cancer cell line (hct-116) and 62.17 µg/mL for regular colon epithelial cell line (FHC) [25]. Valizade et al. also found that *M. eupeus* scorpion venom had an IC_50_ value of 10 μg/mL for the HT-29 colon cancer cell line, while it did not affect the healthy cell line HEK-293 T [26]. Treatment with *M. eupeus* venom significantly reduced the colony number of both cell lines by 52.80 and 63.78%, respectively, compared to the control group **(**Fig. 2c, d**)**. Besides, metastasis potency of the hCRC cells significantly (*p* < 0.001) following the *M. eupeus* venom treatment **(**Fig. 2e, g**)** by 89.24 and 62.28%, respectively **(**Fig. 2f, h**)**. To clarify the death mechanism of the *M. eupeus* venom, the apoptotic and necrotic rates were determined using flow cytometry. After treatment with an equivalent concentration of IC50 values of *M. eupeus* venom, the early apoptotic rate increased by 21.7 and 3.18% in DLD-1 and HT-29 cells, respectively. The late apoptotic rate increased by 3.5 and 10.85% in DLD-1 and HT-29 cell lines, respectively. The necrotic cell rate remained unchanged in both cell lines compared to the control group **(**Fig. 3a, b).

Valizade et al. used MTT assays to evaluate the impact of *M. eupeus* venom on the HT-29 colon cancer cell line [26]. Flow cytometry was used for apoptotic and colony formation analyses. Results showed dose-dependent cytotoxicity with a significant reduction in cell viability at doses above three µg/mL. In addition, flow cytometry analysis indicated that the venom-induced late apoptosis in HT-29 cells compared to early apoptosis [26]. In this study, cytotoxicity studies were performed using the Alamar Blue method, which is more specific and sensitive than MTT. The scorpion venom showed significant cytotoxicity against HT-29 and DLD-1 colon cancer cell lines, while no cytotoxic effect was observed on the colon epithelial cell line CCD18-Co. Apoptosis analysis revealed lower early apoptosis rates in HT-29 and early apoptotic induction in DLD-1 cells. Colony formation experiments showed decreased colony numbers and sizes in HT-29 and DLD-1 cell lines. In silico analyses identified 10 peptides/proteins in *M. eupeus* scorpion venom (Table 1). These peptides were potassium channel inhibitors, suggesting that *M. eupeus* scorpions exert cytotoxic effects by inhibiting K + channels in cells. Furthermore, Gandomkari et al. demonstrated the cytotoxic effect of the recombinant MeICT (rMeICT) peptide isolated from *M. eupeus* venom on glioma cells, with IC50 values of 3 and 5 µM, and in vitro wound healing assays showed wound closure percentages of 58 and 22%, respectively [27]. These findings highlight the potential of rMeICT peptide as an agent targeting gliomas [26]. In this study, in vitro metastasis experiments showed that *M. eupeus* venom inhibited metastasis in both cell lines. However, the HT-29 cell line, with higher adhesive properties, exhibited relatively lower metastasis rates than DLD-1. The different molecular mechanisms and increased protein expressions in the metastatic DLD-1 cells, inhibited by scorpion venom, explain the migration rates between the cell lines.

**Table 1:** *M. eupeus* venom peptides and their possible target channels

Name	Function	Amino acid sequence	Target channels
α-KTx-1.16	Potassium channel toxin blocker	ZFTDVKCTVTKQCWPVCKKMFGRPNGKCMNGKCRCYP	Kv1.1, Kv1.2, Kv1.3, Kv1.6
α-KTx-3.19	Potassium channel toxin blocker	VGINVKCKHSRQCLKPCKDAGMRFGKCMNRKCHCTPK	Kv1.1, Kv1.2, Kv1.3, Kv1.4, Kv1.5, Kv1.6
γ-KTx-2.1	Potassium channel toxin blocker	RPTDIACSESYQCFPVCKSRFGKTNGRCVNGFCDCF	Kv11.1
γ-KTx-2.1	Potassium channel toxin blocker	(R)PTDIKCSESYQCFPVCKSRFGKTNGRCVNGFCDCF	Kv1.3, Kv10.1, Kv11.1
γ-KTx-2.1	Potassium channel toxin blocker	RPTDIKCSESYQCFPVCKSRFGKTNG(K)CVNGFCDCF	Kv1.3, Kv10.1, Kv11.1
α-KTx-32.1	Potassium channel toxin blocker	VDFPNKGGKCDRKECRKTCKKLNYRGKCFNNYCRCFP-NH2	Kv1.1, Kv1.2, Kv1.3, Shaker-IR
β-KTx-2.7	Potassium channel toxin blocker	KMKNSWNRLTSMSEYACPVIEKWCEDHCQAKNAIGRCENTECKCLSK	n.d
α-KTx-1.17	Potassium channel toxin blocker	ZFTDVKCTVTKQCWPVCKKMFGRPNGKCMNGKCRCYS	Kv1.1, Kv1.2, Kv1.3
α-KTx-8.6	Potassium channel toxin blocker	VSCEDCPEHCATKDQRAKCDNDKCVCEPK	Kv1.1,
α-KTx-8.7	Potassium channel toxin blocker	VSCEDCPPHCATKDQRAKCENDKCVCEPK	Kv1.1,

Treatment with *M. eupeus* venom altered the mRNA expressions of genes involved in apoptotic pathways (Figure S1). The venom treatment increased mRNA expression of 19 genes (*ATM*, *BAG3*, *BCL2A1*, *BCL2L11*, *BIRC5*, *BNIP3*, *CASP1*, *CASP12*, *CASP3*, *CIDEB*, *DAPK2*, *DFFB*, *TNF*, *TNFRSF10A*, *TNFRSF10B*, *TNFRSF10C*, *TNFRSF13*, *TNFRSF9*) and decreased mRNA expression of 22 genes (*BCL2*, *BCL2L2*, *BIRC1*, *BIRC2*, *BIRC6*, *BOK*, *BRCC45*, *CARD4*, *CASP8*, *CHEK2*, *FAS*, *GADD45A*, *MCL1*, *MYD88*, *RIPK2*, *RPA3*, *TNFRSF13B*, *TNFRSF15*, *TRAF2*, *TRAF3*, *TRIP*) in apoptotic pathway in DLD-1 cells (Fig. 4a; Table S1). On the other hand, mRNA expression of 12 genes (*APAF1*, *ATM*, *BAG3*, *BAG4*, *BAK1*, *CASP12*, *CASP3*, *CASP5*, *CASP7*, *TRAF3*, *TRAF4*, *TRIP*) was elevated, while 44 gene’s mRNA expression (*BCL2A1*, *BCLX*, *BFAR*, *BIK*, *BIRC1*, *CARD4*, *CASP2*, *CASP6*, *CASP8*, *CASP9*, *CD40L*, *CHEK1*, *CIDEA*, *CIDEB*, *CRADD*, *DAPK1*, *DAPK2*, *DFFA*, *DFFB*, *FAS*, *FASLG*, *GADD45A*, *HRK*, *LTBR*, *MCL1*, *MYD88*, *RIPK2*, *RPA3*, *TANK*, *TNFRSF10*, *TNFRSF10A*, *TNFRSF10B*, *TNFRSF10C*, *TNFRSF11*, *TNFRSF11B*, *TNFRSF13*, *TNFRSF13B*, *TNFRSF15*, *TNFRSF4*, *TNFRSF8*, *TNFRSF9*, *TP53*, *TRAF1*, *TRAF5*) was decreased following venom treatment in HT-29 cells (Fig. 4b; Table S1). mRNA studies have shown that *M. eupeus* venom treatment significantly affects the expression of genes in the apoptotic pathway (Fig. 4c, d). In DLD-1 cells, the mRNA expressions of Bax, Caspase-3, and Caspase-12 significantly increased by 3.7-fold, 12.5-fold, and 1.8-fold, respectively (*p* < 0.0001). In contrast, Bcl-2 mRNA expression decreased by 68.75%. In HT-29 cells, the mRNA expressions of Bax, Caspase-3, Caspase-9, and Caspase-12 significantly increased by 3.01-fold, 2.94-fold, 1.78-fold, and 3.8-fold, respectively (p < 0.0001). Bcl-2 mRNA expression was decreased by 77% compared to non-treated cells.

**Fig. 4:**
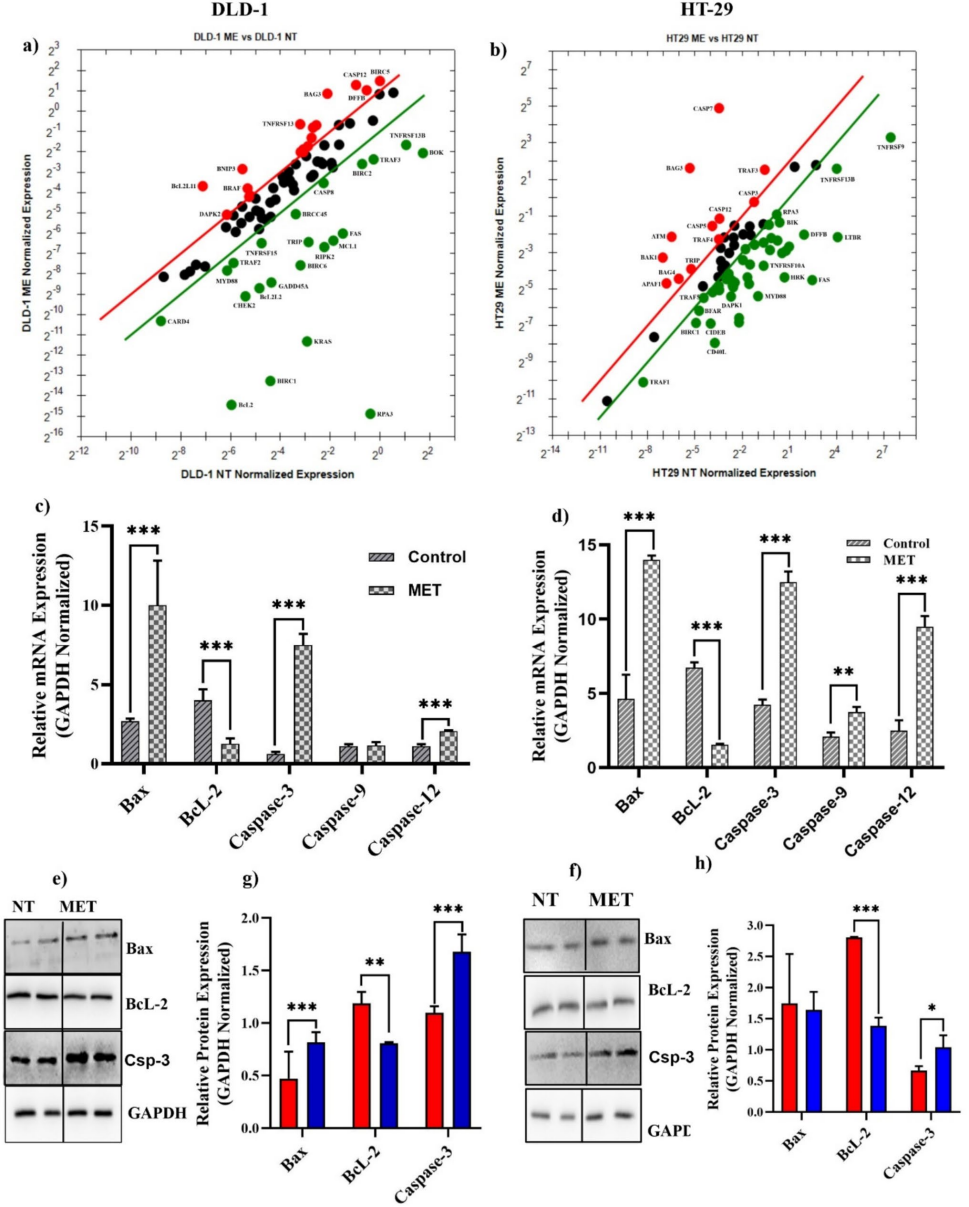
Effects of *M. eupeus* venom on genes and proteins involved in apoptosis. **a**, **b** Alteration in expression levels of 96 genes involved in the apoptosis panel following the treatment with *M. eupeus* scorpion venom. **c**, **d** Alteration in expression levels of genes BcL-2, Bax, caspase-3, caspase-9, and caspase-12, which play an important role in apoptosis, in DLD-1 and HT-29 cell lines compared to the control group. **e**, **f** Representative Immunoblot of proteins Bax, BcL-2, and GAPDH. **g**, **h** Analysis of band density of protein expression of Bax and BcL-2 compared to the control group relative to the reference protein (GAPDH). Results are presented as the mean ± SDV of three independent experiments. *p < 0.01, **p < 0.001, and ***p < 0.0001

Correlated with mRNA expressions, the protein expressions were also altered following the *M. eupeus* venom **(**Fig. 4e, f**)**. The protein expressions of key proteins in the apoptotic pathway, Bax, Bcl-2, and caspase-3, were analyzed **(**Fig. 4g, 4h**)**. In DLD-1 cells, venom treatment significantly (*p* < 0.0001) increased Bax and caspase-3 protein expressions by 1.7-fold and 1.5-fold, respectively. On the other hand, venom treatment decreased Bcl-2 protein expression by 32% (*p* < 0.001) compared with the control group. In HT-29 cells, no alteration was detected in Bax protein expression following venom treatment. However, caspase-3 protein expression was significantly (*p* < 0.05) increased by 1.56-fold, and Bcl-2 protein expression was decreased by 50% (*p* < 0.0001).

**Fig. 5:**
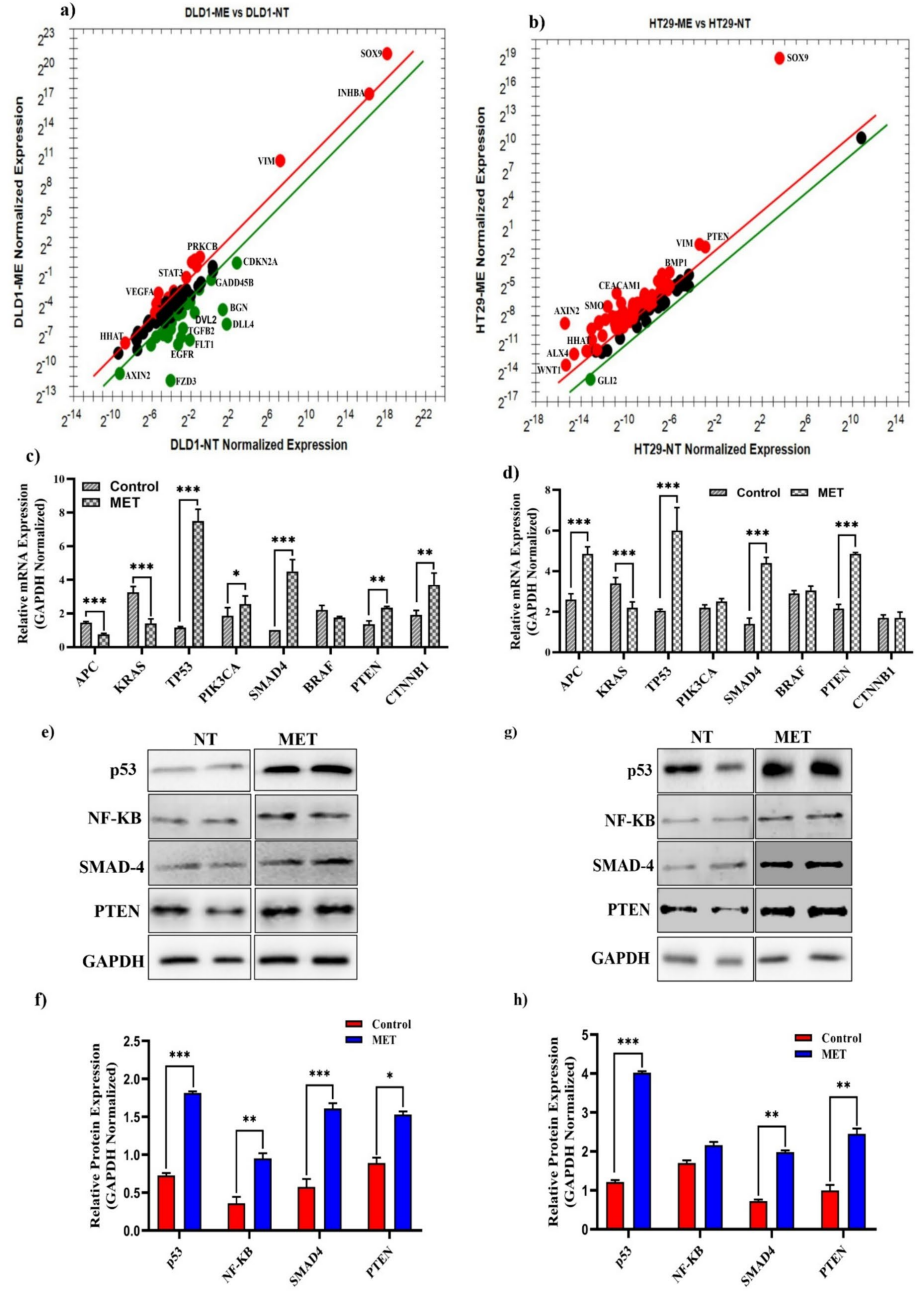
Effects of *M. eupeus* venom on genes and proteins involved in human colorectal carcinoma progression. **a**, **b** Alteration in mRNA expression levels of 96 genes involved in the development of colon cancer following the treatment of *M. eupeus* venom. **c**, **d** Alteration in expression levels of genes NF-κB, TP53, SMAD4, APC, BRAF, KRAS, and MLH-1, which play a role in the human colorectal carcinoma progression, in DLD-1 and HT-29 cell lines compared to the control group. **e**, **g** Representative Immunoblot of NF-κB, P53, and GAPDH proteins. **f**, **h** Analysis of band density of protein expression of NF-κB and P53 compared to the control group relative to the reference protein (GAPDH). Results are presented as the mean ± SDV of three independent experiments. *p < 0.01, and ***p < 0.0001

Treatment with *M. eupeus* venom changed the mRNA expressions of genes involved in human colorectal cancer (hCRC) (Fig. S2). The venom treatment increased mRNA expression of 16 genes (*BMP4*, *CEACAM1*, *HHAT*, *INHBA*, *ITGA4*, *PIK3CA*, *PRKCA*, *PRKCB*, *SMAD4*, *SOX9*, *STAT3*, *TYMS*, *VEGFA*, *VIM*, *WIF1*, *WNT5*) and decreased mRNA expression of 23 genes (*ALX4*, *APC*, *AXIN1*, *AXIN2*, *BGN*, *CDKN2A*, *CEACAM5*, *DLL4*, *DVL2*, *EGFR*, *FLT1*, *FZD1*, *FZD3*, *GADD45B*, *GLI1*, *IGFBP7*, *MAPK3*, *MAPK8*, *MGMT*, *MYC*, *NOTCH2*, *SFRP2*, *TGFB2*) in hCRC progression pathway in DLD-1 cells (Fig. 5a; Table S2). On the other hand, mRNA expression of 57 genes (*BMP4*, *ADAM17*, *AKT1*, *ALX4*, *APC*, *AXIN1*, *AXIN2*, *BGN*, *BMP1*, *BMP2*, *BMP4*, *BMPR1*, *BRAF*, *CDKN2A*, *CDX2*, *CEACAM1*, *CEACAM5*, *CHRD*, *DHH*, *DLL1*, *DLL4*, *DVL1*, *DVL2*, *EGFR*, *FAP*, *FLT1*, *FZD1*, *GADD45B*, *GLI1*, *HHAT*, *IGFBP7*, *ITGA4*, *JAG1*, *JAG2*, *MAPK1*, *MAPK8*, *MYBL2*, *NEUROG1*, *NGFR*, *NOTCH1*, *NOTCH2*, *PRKCA*, *PTCH1*, *PTEN*, *RASSF2*, *SLC5A8*, *SMAD4*, *SMAD3*, *SMAD4*, *SMO*, *SOX9*, *TP53*, *VEGFA*, *VIM*, *WIF1*, *WNT1*) was elevated, while one gene’s mRNA expression (GLI-2) was decreased following venom treatment in HT-29 cells (Fig. 5b; Table S2). mRNA studies have shown that *M. eupeus* venom treatment significantly affects the mRNA expressions of key genes in the hCRC pathway, including *APC*, *KRAS*, *TP53*, *PIK3CA*, *SMAD4*, *BRAF*, *PTEN*, and *CTNNB1* (Fig. 5c; Fig. 5d). In DLD-1 cells, the mRNA expressions of *TP53*, *PIK3CA*, *PTEN*, and *SMAD4* were significantly increased by 6.5-fold, 1.37-fold, 1.7-fold, and 4.5-fold, respectively (*p* < 0.0001), while KRAS mRNA expression decreased by 35.29%. In HT-29 cells, the mRNA expressions of *APC*, *TP53*, *SMAD4*, and *PTEN* significantly increased by 1.86-fold, 2.92-fold, 3.14-fold, and 2.2-fold, respectively (*p* < 0.0001). *Bcl-2* mRNA expression was decreased by 77% compared to non-treated cells. Correlated with the mRNA studies, protein expression studies have also elevated in hCRC (Fig. 5e, f). In DLD-1 cells, venom treatment significantly (*p* < 0.0001) increased p53 protein, SMAD-4, NF-κB, and PTEN protein expressions by 2.6-fold, 3.1-fold, 3.0-fold and 1.8-fold, respectively. Besides, these protein expressions in HT-29 cells were also significantly (*p* < 0.0001) elevated as 3.4-fold, 2.8-fold, 1.2-fold, and 2.6-fold, respectively.

Apoptosis, the process of programmed cell death, plays a crucial role in normal development, tissue maintenance, and the elimination of damaged cells. The apoptosis pathway can be divided into extrinsic and intrinsic pathways. The extrinsic pathway, mediated by death receptors such as Fas, tumor necrosis factor (TNF) receptors, and TNF-related apoptosis-inducing ligand (TRAIL) receptors, is triggered by external or internal stimuli [28]. The binding of ligands to these death receptors activates caspase-8, activating executioner caspases-3/6/7, promoting apoptosis. The decrease in Fas and caspase-8 observed in both cell lines indicates the venom's effect on the apoptosis mechanism [29]. It was demonstrated an increase in caspase-3 and a decrease in Fas and caspase-8 in both DLD-1 and HT-29 cell lines, indicating that the venom induces cell death via the extrinsic apoptosis pathway. In addition, western blot analysis confirmed the increase in caspase-3, further proving the venom's impact on apoptosis.

RIP (Receptor Interacting Protein) and NF-κB are crucial in cell signaling and immune response. RIP is part of a protein complex involved in cell death signaling, and its activation can lead to apoptosis or an inflammatory response during cell stress or damage. NF-κB is a transcription factor that regulates the expression of specific genes. RIP affects the NF-κB signaling pathway, regulating cellular responses such as cell death [30]. Decreased RIP gene expression led to an increase in NF-κB gene expression in both DLD-1 and HT-29 cell lines. Western blot analysis also showed increased NF-κB protein expression in both cell lines, indicating the venom's effect on these proteins. The intrinsic apoptosis pathway is triggered by chemotherapy, radiotherapy, and stress. The functional outcome of pro-apoptotic signaling in the inherent pathway involves mitochondrial membrane disruption and the release of cytochrome c into the cytoplasm, forming the apoptosome with apoptotic protease activating factor 1 (APAF1) and pro-caspase-9. This complex activates initiator caspase-9, which then cleaves and activates caspases-3/6/7, resulting in apoptosis. The apoptosis panel results showed that scorpion venom did not affect intrinsic apoptosis in the DLD-1 cell line. However, increased APAF1 gene expression and decreased caspase-9 gene expression in the HT-29 cell line suggested that the venom induces cell death via intrinsic apoptosis through caspase-3.

The development of colorectal cancer involves chromosomal abnormalities, gene mutations, and epigenetic changes in genes that regulate proliferation, differentiation, apoptosis, and angiogenesis. The Wnt signaling pathway, involving APC and Axin1/2, plays a role in cell proliferation, differentiation, and apoptosis. Overactivation of the Wnt pathway can lead to uncontrolled cancer cell growth and tumor formation [31]. Normally, the Wnt/β-catenin signaling pathway involves APC forming a complex with Axin to degrade the oncogene β-catenin, preventing it from binding to transcription factors in the nucleus and suppressing the expression of genes like c-myc or cyclin D1. In colon cancer, mutated APC cannot form a complex with Axin, allowing β-catenin to activate c-myc or cyclin D1, leading to uncontrolled cell growth. This study showed that APC decreased in the DLD-1 cell line, indicating no effect from the venom, while increases in APC and β-catenin in the HT-29 cell line indicated the venom's impact on the WNT signaling pathway. While this study provides correlative gene expression data suggesting the potential involvement of these pathways, we acknowledge the need for direct experimental validation. The Wnt/β-catenin pathway has been well-documented in colorectal cancer and is known to play a critical role in tumor initiation, progression, and metastasis. Previous studies have shown that aberrant activation of the Wnt/β-catenin pathway can drive cell proliferation, inhibit differentiation, and promote cancer cell survival [32, 33]. The gene expression data of current study suggests that the upregulation of key components of this pathway are consistent with these findings and indicate that this pathway may contribute to the observed effects of *M. eupeus* venom. Similarly, NF-κB signaling is a central regulator of inflammation, immune responses, apoptosis, and cancer progression. Dysregulation of NF-κB has been linked to colorectal cancer pathogenesis, chemoresistance, and metastasis [34]. In particular, studies have shown that the activation of NF-κB can promote tumor growth and resistance to chemotherapy in colorectal cancer [35]. This study’s gene expression analysis aligns with these observations, suggesting the potential involvement of NF-κB signaling in the cytotoxic effects induced by the venom. TP53 is a tumor suppressor gene that increases in response to DNA damage, halting cell division, inducing apoptosis and senescence, and facilitating DNA repair by binding to the promoter regions of genes involved in these processes [36]. The increase in TP53 gene expression was further confirmed that the venom induces cell death via extrinsic apoptosis in DLD-1 and intrinsic and extrinsic apoptosis in HT-29 cell lines. In the TGFβ signaling pathway, Smad4 is crucial in promoting metastasis and proliferation in colon cancer cells [37]. Tumor suppressor Smad proteins are activated upon binding to TGFβ receptors and translocate to the nucleus to regulate the expression of specific genes, thereby inhibiting metastasis and uncontrolled cell proliferation [38]. It has been demonstrated increased gene expression of Smad4 in both DLD-1 and HT-29 cell lines. This indicates that *M. eupeus* scorpion venom inhibits cell metastasis and growth. KRAS is a gene and protein complex commonly known as an oncogene called Kirsten Rat Sarcoma Virus Proto-Oncogene. The KRAS gene stimulates cellular growth and division by binding to receptors on the cell surface. When mutated, the KRAS gene can lead to uncontrolled cellular growth and division, contributing to the development of cancerous tumors [39]. It has observed a decreased KRAS gene expression in DLD-1 and HT-29 cell lines. This suggests that *M. eupeus* scorpion venom inhibits cell growth. The BRAF gene is a proto-oncogene involved in the MAPK (Mitogen-Activated Protein Kinase) signaling pathway, which regulates intracellular signal transduction. This pathway is crucial for cellular growth, proliferation, differentiation, and survival. Mutations in the BRAF gene can lead to the overactivation of the MAPK signaling pathway, resulting in uncontrolled cell growth and division [40]. *M. eupeus* scorpion venom did not significantly affect the BRAF gene. PIK3CA, a tumor suppressor gene, encodes the catalytic alpha subunit of phosphatidylinositol 3-kinase (PI3K). This gene is a key component of the PI3K/AKT/mTOR signaling pathway, which regulates cell growth, proliferation, differentiation, survival, and metabolism. Overactivation or dysregulation of this pathway plays a role in cancer development [41]. *M. eupeus* scorpion venom increased the expression level of this gene in the DLD-1 cell line but did not affect the HT-29 cell line. PTEN is a tumor suppressor gene and protein complex involved in cell growth, proliferation, survival, and metabolism. The PTEN gene controls cell growth by regulating intracellular signal transduction. Mutations in the PTEN gene or the functional loss of the PTEN protein can lead to uncontrolled cell growth and division, contributing to the development of cancerous tumors [42]. Our studies have found that *M. eupeus* scorpion venom activates this gene in DLD-1 and HT-29 cell lines, preventing cancer formation. CTNNB1 is associated with the beta-catenin protein, which regulates cell adhesion and communication. Mutations in the CTNNB1 gene or abnormal activation of the beta-catenin protein can lead to the overactivation of several signaling pathways that regulate cell growth and division, contributing to cancer development and progression [43]. This study observed that *M. eupeus* scorpion venom activated this tumor suppressor gene in the DLD-1 cell line, while no change was observed in the HT-29 cell line. The SOX9 gene, a transcription factor, can act as either a proto-oncogene or a tumor suppressor gene, depending on the type of cancer. SOX9 regulates the tumor microenvironment, maintains epithelial barrier integrity, and preserves undifferentiated stem cells for tissue renewal in the intestinal epithelium. Studies have shown that the overexpression of SOX9 in colon cancer cells inhibits cell proliferation, while a decrease in SOX9 expression increases the proliferation of human HT-29 colon cancer cells [44]. The studies have found that SOX9 acts as a tumor suppressor gene, with increased expression levels in both DLD-1 and HT-29 cell lines. Additionally, SOX9 influences cell migration and invasion through the Wnt/β-catenin signaling pathway [45]. GLI-2, a prognostic marker in colon cancer, showed decreased expression in both cell lines [46]. Similarly, VIM, a gene expressed at high levels in metastatic tumors, showed increased expression in DLD-1 and HT-29 cell lines [47].

The conformation of a chemical agent to a drug candidate takes an extended period due to the toxic nature of the compound [48]. Captopril, ziconotide, atracurium, and eptifibatide are FDA-proven drugs formulated from venom toxins [49]. Vejovine is also an antimicrobial peptide purified from the venom of the scorpion *V. mexicanus smithi*. This peptide also showed hemolytic activity against human erythrocytes with 100 mM HC_50_ [50]. The molecular studies proved that *M. eupeus* venom has the potential to deal with human colorectal carcinoma. Hence, the active compounds in the venom may be further investigated for their potential against hCRC.

## Conclusions

In conclusion, *M. eupeus* scorpion venom demonstrates promising potential as a novel therapeutic approach for colon cancer treatment, showing significant cytotoxic effects on cancer cell lines. Our study reveals its capacity to induce apoptosis, inhibit colony formation, and modulate critical signaling pathways, including *TGFβ*, *Wnt,* and *mTOR-NF-κB. It highlights* its multifaceted role in regulating proliferation, metastasis, and cellular behavior. The venom’s selective cytotoxicity towards cancer cells aligns with previous studies, underscoring its potential as a targeted therapeutic agent. Despite the promising in vitro findings, this study has several limitations, primarily the reliance on cell culture models, which do not fully replicate the complexity of in vivo systems. Future studies must address these limitations and elucidate the venom’s precise molecular mechanisms. In particular, compound isolation and in vivo models are critical next steps to assess the venom's therapeutic efficacy, safety, and potential for clinical translation. While this research serves as an essential step in understanding the venom's therapeutic potential, the following phase should focus on isolating the active components, validating their effects in animal models, and evaluating their safety profile. The promising results of this study provide a solid foundation for future work in this area, with *M. eupeus* venom emerging as a compelling candidate for the development of innovative, natural cancer therapies.

## Material and methods

### Collection of scorpions and extraction of venoms

*M. eupeus* were collected at night from Iğdır Province (Melekli Village, 5 km north-east, 39°57′30′′ N, 44°08′42′′, 865 m, leg. Yağmur, Sipahioğlu and Kartal) in 16.07.2022. Scorpions were housed in 5-L containers with cocopeat soil and an egg container for shelter. Venom was extracted after carbon dioxide anesthesia, using 12 V/25A electrical stimulation. Processed venom was diluted (0.85% NaCl), lyophilized, and stored at − 20 °C.

### Characterization studies of *Mesobuthus eupeus* scorpion venom

To determine the maximum peak of venom, the powdered *M. eupeus* venom, prepared at a concentration of 1 mg/mL, was analyzed using a UV–VIS spectrophotometer across the wavelength range of 190 nm to 400 nm. The crude lyophilized venom (2 mg) was dissolved in 200 μL ultrapure water and subjected to C-18 reverse-phase high-performance liquid chromatography (HPLC). The C-18 column was initially equilibrated with 0.1% trifluoroacetic acid (TFA) in water (Eluent A) and with 0.1% TFA in acetonitrile (ACN) (Eluent B) using a linear gradient at a rate of 1 mL/min. The resulting peaks were monitored at 280 nm using a PDA detector.

### Determination of concentration and fractions of the venom

The total protein concentration of the venom was determined using the bicinchoninic acid (BCA) method, and BSA was used as the standard [51]. Electrophoretic separation of *M. eupeus* venom was performed using sodium dodecyl sulfate–polyacrylamide gel electrophoresis (SDS-PAGE) [52]. 5–30 µg of lyophilized venom was loaded onto a 10% SDS-PAGE gel under non-reducing conditions. A constant current of 20 mA was applied to the gel in a running buffer containing 25 mM Tris–glycine, 192 mM glycine, and 0.1% SDS at pH 8.8. The gel was then stained with 0.2% Coomassie Brilliant Blue R-250 for 30 min and destained using a solvent composed of methanol, acetic acid, and distilled water in a ratio of 25:12.5:62.5. Finally, the protein bands were visualized and captured using a Syngene Imaging System.

### Cell culture conditions

Human colon cancer cell lines DLD-1 and HT-29 and healthy colon cell line CCD-18Co were cultured in RPMI-1640, McCoy's 5A Medium, and EMEM at 37 °C with 5% CO_2_ in an incubator. The growth medium was supplemented with 10% FBS, 1% penicillin–streptomycin, and two mM l-glutamine to support cell growth and maintenance.

### Cell viability and cytotoxicity studies

To assess the impact of scorpion venom on cell proliferation, human colon cancer cells, and healthy epithelial cells were seeded onto 96-well plates at a density of 1 × 10^4^ cells per well. Following 24-h incubation, the cells were exposed to varying concentrations (0–250 µg/mL) of scorpion venom and incubated. Subsequently, cells were treated with Alamar blue reagent for 3 h as described previously [53]. A sigmoidal curve was plotted from cell viability data, and the concentration of scorpion venom that inhibited 50% of cell growth (IC_50_) was calculated.

### Cell migration assay

To investigate the impact of *M. eupeus* scorpion venom on metastatic properties, the CytoSelect Wound Healing Assay (CellBiolabs, USA) was conducted. The cells were seeded onto 6-well plates, treated with an equivalent concentration of the venom IC50 value, and incubated for 48 h at 37 °C with 5% CO_2_. Then, the cells were trypsinized and transferred into the inserts, which were placed into 24-well plates. Cells were seeded onto the inserts at a density of 1 × 10^5^ cells and left for a 24-h incubation period. Post-incubation, the inserts were removed, and images of both the control and venom-treated groups were captured at 12-h intervals until wound closure. The quantification of migrated cells was performed using Image J software.

### Colony formation assay

A soft-agar colony formation assay was conducted to investigate the effects of *M. eupeus* scorpion venom on the cellular colony-forming properties of colorectal carcinoma cells. The cells were treated with an equivalent concentration of the venom's IC_50_ value and incubated for 48 h at 37 °C with 5% CO_2_. Subsequently, a 10% bottom agar layer was spread on the base of a 6-well plate. 5 × 10^3^ cells were mixed with 0.7% top agar and layered over the bottom agar. After gel solidification, the cells’ growth medium was added to the plate, which was then incubated at 37 °C with 5% CO_2_ for 15 days. Post-incubation, cells were stained with 1% Crystal Violet, and colony quantification was performed under an inverted microscope using Image J software.

### Apoptosis studies

Using Annexin V-APC/ 7-AAD apoptosis kit (eBioscience, USA), the effects of *M. eupeus* venom on cell death mechanisms were determined by flow cytometry. The cells were treated with an equivalent concentration of the venom's IC_50_ value and incubated for 48 h at 37 °C with 5% CO_2_. Then, the cells were centrifuged at 15.000×*g* for 5 min. The pellet was stained with Annexin V-APC and 7-AAD antibodies according to the apoptosis kit protocol (BD Biosciences, San Jose, CA, USA).

### mRNA expression analysis of apoptotic and colon cancer progression pathways

Quantitative real-time polymerase chain reaction (qRT-PCR) studies were conducted to elucidate alterations in gene expressions of apoptotic and CRC pathways induced by *M. eupeus* venom. The cells were treated with an equivalent concentration of the venom's IC_50_ value and incubated for 24 h at 37 °C with 5% CO_2_. Following treatment, total RNA was isolated using the Trizol method. The purity and quantity of the obtained RNA were measured using the NanoDrop™ 2000 (Thermo Scientific). iScript cDNA Synthesis Kit (Bio-Rad, USA) was employed to synthesize cDNA. The Human Colorectal Cancer Panel and Human Apoptosis Panel (Figs. S1, Fig. S2) were used to investigate the alteration in mRNA expressions of the apoptotic pathway and CRC progression signaling pathway using quantitative real-time PCR (qRT-PCR). The relative mRNA expression was calculated using the 2^−ΔΔCt^ method. *GAPDH*, *GUSB*, *PPIA*, *B2M*, *HPRT1*, *PGK1*, *ACTB,* and *RPL13A* were used as internal standards. Both panels were designed using Primer 3 software and verified for gene specificity using NCBI blast software (Table S3).

### Protein expression analysis of apoptotic pathway and colon cancer progression proteins

Western blot studies were conducted to assess the effects of *M. eupeus* venom on protein expressions related to hCRC and apoptosis. Cells were treated with total scorpion venom at the IC_50_ value and incubated for 48 h at 37 °C with 5% CO_2_. SDS-PAGE separated the lysate on 7.5–12% resolving gels based on the target protein's molecular weight. The separated proteins were transferred to a PVDF membrane. After membrane transfer, the bands were prepared for antibody binding. Primary antibodies for Bax (21 kDa, 1/1000 dilution), Bcl-2 (26 kDa, 1/1000 dilution) MLH-1 (85 kDa,1/500 dilution), p53 (53 kDa, 1/1000 dilution), NF-κB (65 kDa, 1/1000 dilution), and GAPDH (36 kDa,1/2000 dilution) were incubated overnight at 4 °C. The following day, the membranes were incubated with secondary antibodies at room temperature for 1 h. Protein bands were visualized using an ECL solution (Bio-Rad, USA) and captured using a Chemiluminescent Gel Documentation System. The density of protein bands was analyzed using the GeneSys imaging software.

### Statistical analysis

Statistical analyses of the experimental results were conducted using GraphPad Prism 8.0 software. Comparisons between two groups were performed using the Student's t-test, while within-group comparisons were made using ANOVA. A significance level of *p* < 0.05 was considered statistically significant. All experiments were independently repeated three (3) times.

## Electronic supplementary material

Below is the link to the electronic supplementary material.

Supplementary file1 (DOCX 289 KB)

## Data Availability

Data will be made available on request. No datasets were generated or analyzed during the current study.
