# Supplementary material for: Mesobuthus eupeus venom modulates colorectal carcinoma signaling pathways and induces apoptosis
Source: Med Oncol. 2025 Apr 14;42(5):163. doi: 10.1007/s12032-025-02689-2 (PMC11996983; doi:10.1007/s12032-025-02689-2)
Supplement: Supplementary file 1 — Supplementary file1 (DOCX 289 KB) [file 12032_2025_2689_MOESM1_ESM.docx]

***Mesobuthus eupeus* Venom Modulates Colorectal Carcinoma Signaling Pathways and Induces Apoptosis**

Havva Nur CANAK^1^ **·**Kemal BAS^1^ **·**Ersen Aydın YAĞMUR^2^ **·**Serdar KARAKURT^1, *^

^1^Faculty of Science, Department of Biochemistry, Selcuk University, Konya, Türkiye

^2^Alasehir Vocational High School, Manisa Celal Bayar University, Manisa, Türkiye

**^*^Corresponding Author:** Prof. Dr. Serdar KARAKURT

**e-mail:** kserdar1@yahoo.com

<https://orcid.org/> 0000-0002-4449-610

Department of Biochemistry, Faculty of Science, Selcuk University, Konya, Türkiye

**SUPPLEMENTARY DATA**

**
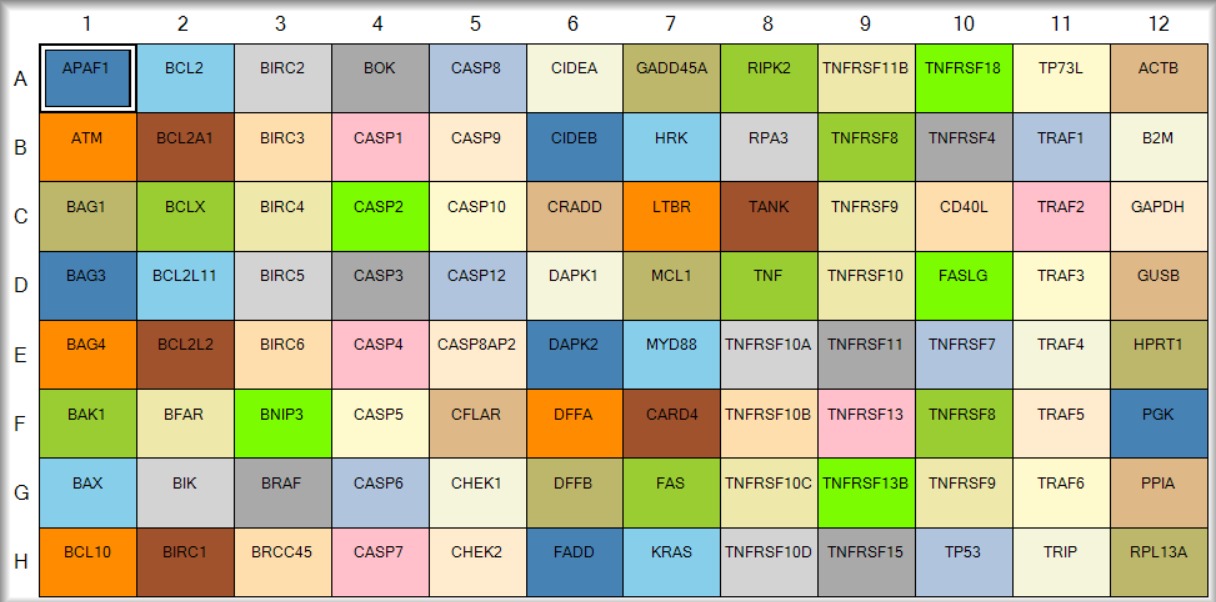
**

**Fig. S1.** Real-time PCR panel for Apoptosis: The mRNA expression of 88 genes involved in apoptosis was investigated. GAPDH, GUSB, PPIA, B2M, HPRT1, PGK1, ACTB, and RPL13A were used as internal standards.

**
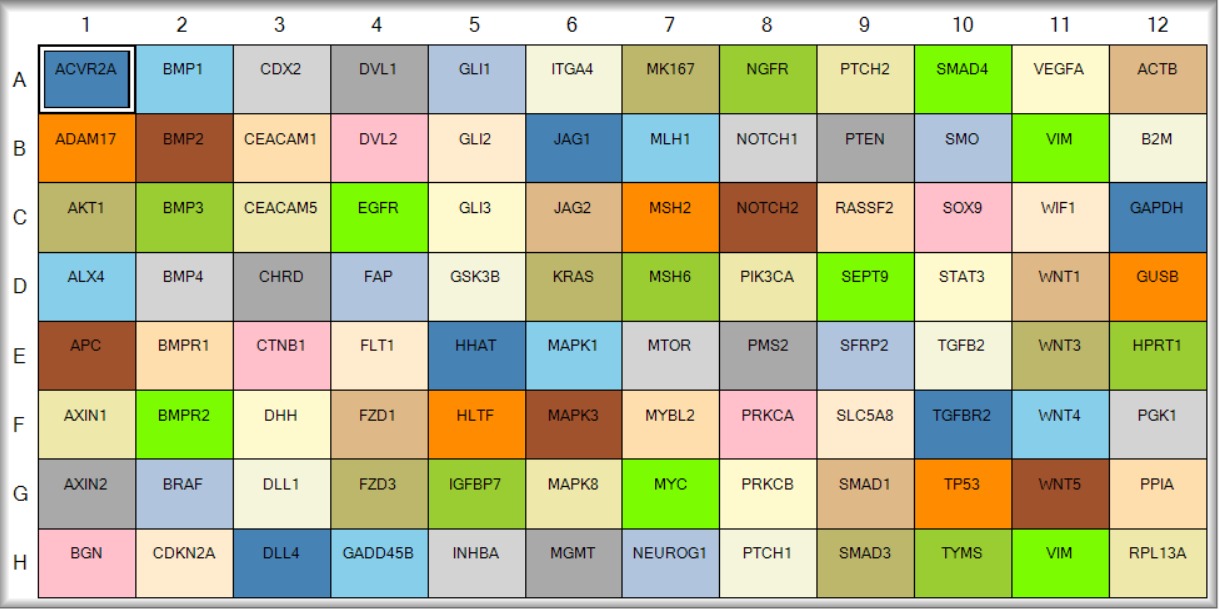
**

**Fig. S2.** Real-time PCR panel for Human Colorectal Carcinoma: The mRNA expression of 88 genes involved in apoptosis was investigated. *GAPDH, GUSB, PPIA, B2M, HPRT1, PGK1, ACTB,* and *RPL13A* were used as internal standards.

**Table S1.** Comparison of Gene Expression in Apoptosis Pathway Between DLD-1 and HT-29 Cells

| **GENE SYMBOL** | **GENE NAME** | **GENE EXPRESSION** | |
| --- | --- | --- | --- |
|  |  | **DLD-1** | **HT-29** |
| **APAF1** | Apoptotic Peptidase Activating Factor 1 | no change | up-regulated |
| **ATM** | ATM Serine/Threonine Kinase | up-regulated | up-regulated |
| **BAG1** | BAG Cochaperone 1 | no change | no change |
| **BAG3** | BAG Cochaperone 3 | up-regulated | up-regulated |
| **BAG4** | BAG Cochaperone 4 | no change | up-regulated |
| **BAK1** | BCL2 Antagonist/Killer 1 | no change | up-regulated |
| **BAX** | BCL2 Associated X, Apoptosis Regulator | no change | no change |
| **BCL10** | BCL10 İmmune Signaling Adaptor | no change | no change |
| **BCL2** | BCL2 Apoptosis Regulator | down-regulated | no change |
| **BCL2A1** | BCL2 Related Protein A1 | up-regulated | down-regulated |
| **BCLX (BCL2L1)** | BCL2 Like 1 | no change | down-regulated |
| **BCL2L11** | BCL2 Like 11 | up-regulated | no change |
| **BCL2L2** | BCL2 Like 2 | down-regulated | no change |
| **BFAR** | Bifunctional Apoptosis Regulator | no change | down-regulated |
| **BIK** | BCL2 İnteracting Killer | no change | down-regulated |
| **BIRC1** | Baculoviral IAP Repeat Containing 1 | down-regulated | down-regulated |
| **BIRC2** | Baculoviral IAP Repeat Containing 2 | down-regulated | no change |
| **BIRC3** | Baculoviral IAP Repeat Containing 3 | no change | no change |
| **BIRC4** | Baculoviral IAP Repeat Containing 4 | no change | no change |
| **BIRC5** | Baculoviral IAP Repeat Containing 5 | up-regulated | no change |
| **BIRC6** | Baculoviral IAP Repeat Containing 6 | down-regulated | no change |
| **BNIP3** | BCL2 İnteracting Protein 3 | up-regulated | no change |
| **BRAF** | B-Raf Proto-Oncogene, Serine/Threonine Kinase | up-regulated | no change |
| **BRCC45 (BABAM2)** | BRISC And BRCA1 A Complex Member 2 | down-regulated | no change |
| **BOK** | BCL2 Family Apoptosis Regulator BOK | down-regulated | no change |
| **CASP1** | Caspase 1 | up-regulated | no change |
| **CASP2** | Caspase 2 | no change | down-regulated |
| **CASP3** | Caspase 3 | up-regulated | up-regulated |
| **CASP4** | Caspase 4 | no change | no change |
| **CASP5** | Caspase 5 | no change | up-regulated |
| **CASP6** | Caspase 6 | no change | down-regulated |
| **CASP7** | Caspase 7 | no change | up-regulated |
| **CASP8** | Caspase 8 | down-regulated | down-regulated |
| **CASP9** | Caspase 9 | no change | down-regulated |
| **CASP10** | Caspase 10 | no change | no change |
| **CASP12** | Caspase 12 | up-regulated | up-regulated |
| **CASP8AP2** | Caspase 8 Associated Protein 2 | no change | no change |
| **CFLAR** | CASP8 And FADD Like Apoptosis Regulator | no change | no change |
| **CHEK1** | Checkpoint Kinase 1 | no change | down-regulated |
| **CHEK2** | Checkpoint Kinase 2 | down-regulated | no change |
| **CIDEA** | Cell Death İnducing DFFA Like Effector A | no change | down-regulated |
| **CIDEB** | Cell Death İnducing DFFA Like Effector B | up-regulated | down-regulated |
| **CRADD** | CASP2 And RIPK1 Domain Containing Adaptor With Death Domain | no change | down-regulated |
| **DAPK1** | Death Associated Protein Kinase 1 | no change | down-regulated |
| **DAPK2** | Death Associated Protein Kinase 2 | up-regulated | down-regulated |
| **DFFA** | DNA Fragmentation Factor Subunit Alpha | no change | down-regulated |
| **DFFB** | DNA Fragmentation Factor Subunit Beta | up-regulated | down-regulated |
| **FADD** | Fas Associated Via Death Domain | no change | no change |
| **GADD45A** | Growth Arrest And DNA Damage İnducible Alpha | down-regulated | down-regulated |
| **HRK** | Harakiri, BCL2 İnteracting Protein | no change | down-regulated |
| **LTBR** | Lymphotoxin Beta Receptor | no change | down-regulated |
| **MCL1** | MCL1 Apoptosis Regulator, BCL2 Family Member | down-regulated | down-regulated |
| **MYD88** | MYD88 İnnate İmmune Signal Transduction Adaptor | down-regulated | down-regulated |
| **CARD4 (NOD1)** | Nucleotide Binding Oligomerization Domain Containing 1 | down-regulated | down-regulated |
| **FAS** | Fas Cell Surface Death Receptor | down-regulated | down-regulated |
| **KRAS** | KRAS Proto-Oncogene, Gtpase | down-regulated | no change |
| **RIPK2** | Receptor İnteracting Serine/Threonine Kinase 2 | down-regulated | down-regulated |
| **RPA3** | Replication Protein A3 | down-regulated | down-regulated |
| **TANK** | TRAF Family Member Associated NFKB Activator | no change | down-regulated |
| **TNF** | Tumor Necrosis Factor | up-regulated | no change |
| **TNFRSF10A** | TNF Receptor Superfamily Member 10a | up-regulated | down-regulated |
| **TNFRSF10B** | TNF Receptor Superfamily Member 10b | up-regulated | down-regulated |
| **TNFRSF10C** | TNF Receptor Superfamily Member 10c | up-regulated | down-regulated |
| **TNFRSF10D** | TNF Receptor Superfamily Member 10d | no change | no change |
| **TNFRSF11B** | TNF Receptor Superfamily Member 11b | no change | down-regulated |
| **TNFRSF8** | TNF Receptor Superfamily Member 8 | no change | no change |
| **TNFRSF9** | TNF Receptor Superfamily Member 9 | up-regulated | no change |
| **TNFRSF10** | TNF Receptor Superfamily Member 10 | no change | no change |
| **TNFRSF11** | TNF Receptor Superfamily Member 11 | no change | down-regulated |
| **TNFRSF13** | TNF Receptor Superfamily Member 13 | up-regulated | down-regulated |
| **TNFRSF13B** | TNF Receptor Superfamily Member 13b | down-regulated | down-regulated |
| **TNFRSF15** | TNF Receptor Superfamily Member 15 | down-regulated | down-regulated |
| **TNFRSF18** | TNF Receptor Superfamily Member 18 | no change | no change |
| **TNFRSF4** | TNF Receptor Superfamily Member 4 | no change | down-regulated |
| **CD40L** | CD40 Ligand | no change | down-regulated |
| **FASLG** | Fas Ligand | no change | down-regulated |
| **TNFRSF7** | TNF Receptor Superfamily Member 7 | no change | no change |
| **TNFRSF8** | TNF Receptor Superfamily Member 8 | no change | down-regulated |
| **TNFRSF9** | TNF Receptor Superfamily Member 9 | no change | down-regulated |
| **TP53** | Tumor Protein P53 | no change | down-regulated |
| **TP73L** | Tumor Protein P73 | no change | no change |
| **TRAF1** | TNF Receptor Associated Factor 1 | no change | down-regulated |
| **TRAF2** | TNF Receptor Associated Factor 2 | down-regulated | no change |
| **TRAF3** | TNF Receptor Associated Factor 3 | down-regulated | up-regulated |
| **TRAF4** | TNF Receptor Associated Factor 4 | no change | up-regulated |
| **TRAF5** | TNF Receptor Associated Factor 5 | no change | down-regulated |
| **TRAF6** | TNF Receptor Associated Factor 6 | no change | no change |
| **TRIP** | Thyroid Hormone Receptor İnteractor | down-regulated | up-regulated |

**Table S2.** Comparison of Gene Expression in Apoptosis Pathway Between DLD-1 and HT-29 Cells

| **GENE SYMBOL** | **GENE NAME** | **GENE EXPRESSION** | |
| --- | --- | --- | --- |
|  |  | **DLD-1** | **HT-29** |
| **ACVR2A** | Activin A Receptor, Type IIA | no change | no change |
| **ADAM17** | ADAM Metallopeptidase Domain 17 | no change | up-regulated |
| **AKT1** | V-Akt Murine Thymoma Viral Oncogene Homolog 1 | no change | up-regulated |
| **ALX4** | ALX Homeobox 4 | down-regulated | up-regulated |
| **APC** | Adenomatous Polyposis Coli | down-regulated | up-regulated |
| **AXIN1** | Axin 1 | down-regulated | up-regulated |
| **AXIN2** | Axin 2 | down-regulated | up-regulated |
| **BGN** | Biglycan | down-regulated | up-regulated |
| **BMP1** | Bone Morphogenetic Protein 1 | no change | up-regulated |
| **BMP2** | Bone Morphogenetic Protein 2 | no change | up-regulated |
| **BMP3** | Bone Morphogenetic Protein 3 | no change | no change |
| **BMP4** | Bone Morphogenetic Protein 4 | up-regulated | up-regulated |
| **BMPR1** | Bone Morphogenetic Protein Receptor, Type I | no change | up-regulated |
| **BMPR2** | Bone Morphogenetic Protein Receptor, Type II | no change | no change |
| **BRAF** | B-Raf Proto-Oncogene, Serine/Threonine Kinase | no change | up-regulated |
| **CDKN2A** | Cyclin-Dependent Kinase İnhibitor 2A | down-regulated | up-regulated |
| **CDX2** | Caudal Type Homeobox 2 | no change | up-regulated |
| **CEACAM1** | Carcinoembryonic Antigen-Related Cell Adhesion Molecule 1 (Biliary Glycoprotein) | up-regulated | up-regulated |
| **CEACAM5** | Carcinoembryonic Antigen-Related Cell Adhesion Molecule 5 | down-regulated | up-regulated |
| **CHRD** | Chordin | no change | up-regulated |
| **CTNB1** | Catenin (Cadherin-Associated Protein), Beta 1, 88kda | no change | no change |
| **DHH** | Desert Hedgehog | no change | up-regulated |
| **DLL1** | Delta-Like 1 (Drosophila) | no change | up-regulated |
| **DLL4** | Delta-Like 4 (Drosophila) | down-regulated | up-regulated |
| **DVL1** | Dishevelled Segment Polarity Protein 1 | no change | up-regulated |
| **DVL2** | Dishevelled Segment Polarity Protein | down-regulated | up-regulated |
| **EGFR** | Epidermal Growth Factor Receptor | down-regulated | up-regulated |
| **FAP** | Fibroblast Activation Protein, Alpha | no change | up-regulated |
| **FLT1** | Fms-Related Tyrosine Kinase 1 | down-regulated | up-regulated |
| **FZD1** | Frizzled Class Receptor 1 | down-regulated | up-regulated |
| **FZD3** | Frizzled Class Receptor 3 | down-regulated | no change |
| **GADD45B** | Growth Arrest And DNA-Damage-İnducible, Beta | down-regulated | up-regulated |
| **GLI1** | GLI Family Zinc Finger 1 | down-regulated | up-regulated |
| **GLI2** | GLI Family Zinc Finger 2 | no change | down-regulated |
| **GLI3** | GLI Family Zinc Finger 3 | no change | no change |
| **GSK3B** | Glycogen Synthase Kinase 3 Beta | no change | no change |
| **HHAT** | Hedgehog Acyltransferase | up-regulated | up-regulated |
| **HLTF** | Helicase-Like Transcription Factor | no change | no change |
| **IGFBP7** | İnsulin-Like Growth Factor Binding Protein 7 | down-regulated | up-regulated |
| **INHBA** | İnhibin, Beta A | up-regulated | no change |
| **ITGA4** | İntegrin, Alpha 4 (Antigen CD49D, Alpha 4 Subunit Of VLA-4 Receptor) | up-regulated | up-regulated |
| **JAG1** | Jagged 1 | no change | up-regulated |
| **JAG2** | Jagged 2 | no change | up-regulated |
| **KRAS** | Kirsten Rat Sarcoma Viral Oncogene Homolog | no change | up-regulated |
| **MAPK1** | Mitogen-Activated Protein Kinase 1 | no change | up-regulated |
| **MAPK3** | Mitogen-Activated Protein Kinase 3 | down-regulated | no change |
| **MAPK8** | Mitogen-Activated Protein Kinase 8 | down-regulated | up-regulated |
| **MGMT** | O-6-Methylguanine-DNA Methyltransferase | down-regulated | no change |
| **MKI67** | Marker Of Proliferation Ki-67 | no change | no change |
| **MLH1** | Mutl Homolog 1 | no change | no change |
| **MSH2** | Muts Homolog 2 | no change | no change |
| **MSH6** | Muts Homolog 6 | no change | no change |
| **MTOR** | Mechanistic Target Of Rapamycin (Serine/Threonine Kinase) | no change | no change |
| **MYBL2** | V-Myb Avian Myeloblastosis Viral Oncogene Homolog- Like 2 | no change | up-regulated |
| **MYC** | V-Myc Avian Myeloblastosis Viral Oncogene Homolog | down-regulated | no change |
| **NEUROG1** | Neurogenin 1 | no change | up-regulated |
| **NGFR** | Nerve Growth Factor Receptor NOG Noggin | no change | up-regulated |
| **NOTCH1** | Notch 1 | no change | up-regulated |
| **NOTCH2** | Notch 2 | down-regulated | up-regulated |
| **PIK3CA** | Phosphatidylinositol-4,5-Bisphosphate 3-Kinase, Catalytic Subunit Alpha | up-regulated | no change |
| **PMS2** | PMS2 Postmeiotic Segregation İncreased 2 (S. Cerevisiae) | no change | no change |
| **PRKCA** | Protein Kinase C, Alpha | up-regulated | up-regulated |
| **PRKCB** | Protein Kinase C, Beta | up-regulated | no change |
| **PTCH1** | Patched 1 | no change | up-regulated |
| **PTCH2** | Patched 2 | no change | no change |
| **PTEN** | Phosphatase And Tensin Homolog | no change | up-regulated |
| **RASSF2** | Ras Association (Ralgds/AF-6) Domain Family Member 2 | no change | up-regulated |
| **SEPT9** | Septin 9 | no change | no change |
| **SFRP2** | Secreted Frizzled-Related Protein 2 | down-regulated | no change |
| **SLC5A8** | Solute Carrier Family 5 (Sodium/Monocarboxylate Cotransporter), Member 8 | no change | up-regulated |
| **SMAD1** | SMAD Family Member 1 | no change | up-regulated |
| **SMAD3** | SMAD Family Member 3 | up-regulated | up-regulated |
| **SMAD4** | SMAD Family Member 4 | no change | up-regulated |
| **SMO** | Smoothened, Frizzled Class Receptor | no change | up-regulated |
| **SOX9** | SRY (Sex Determining Region Y)-Box 9 | up-regulated | up-regulated |
| **STAT3** | Signal Transducer And Activator Of Transcription 3 (Acute-Phase Response Factor) | up-regulated | no change |
| **TGFB2** | Transforming Growth Factor, Beta 2 | down-regulated | no change |
| **TGFBR2** | Transforming Growth Factor, Beta Receptor II (70/80kda) | no change | no change |
| **TP53** | Tumor Protein P53 | no change | up-regulated |
| **TYMS** | Thymidylate Synthetase | up-regulated | no change |
| **VEGFA** | Vascular Endothelial Growth Factor A | up-regulated | up-regulated |
| **VIM** | Vimentin | up-regulated | up-regulated |
| **WIF1** | WNT İnhibitory Factor 1 | up-regulated | up-regulated |
| **WNT1** | Wingless-Type MMTV İntegration Site Family, Member 1 | no change | up-regulated |
| **WNT3** | Wingless-Type MMTV İntegration Site Family, Member 3 | no change | no change |
| **WNT4** | Wingless-Type MMTV İntegration Site Family, Member 4 | no change | no change |
| **WNT5** | Wingless-Type MMTV İntegration Site Family, Member 5 | up-regulated | no change |
| **VIM** | Vimentin | no change | no change |

**Table S3.** Sequence and Annealing Temperature of the Primers

| **Primer Name** | **Primer Sequence(5‘→3’)*** | **Annealing Temperature (°C)** |
| --- | --- | --- |
| APC | F- TGAGGCACTGAAGATGGAGA  R- TCTGTCCAGAAGAAGCCATAG | 56 |
| PIK3CA | F- TATGGTTGTCTGTCAATCGGTGA  R- GCCTTTGCAGTGAATTTGCAT | 57 |
| TP53 | F- CACATGACGGAGGTTGTGAG  R- TAGGGCACCACCACACTATG | 58 |
| PTEN | F-TGAGTTCCCTCAGCCGTTACCT  R-GAGGTTTCCTCTGGTCCTGGTA | 58 |
| CTNNB1 | F-GAACTGTCTTTGGACTCTCAGG  R-TGCACAGGTGACCACATTTA | 59 |
| SMAD4 | F- CACAGGACAGAAGCCATTGA  R- ACGCCCAGCTTCTCTGTCTA | 57 |
| KRAS | F- TGCAATGAGGGACCAGTACA  R- TCCTGAGCCTGTTTTGTGTCT | 58 |
| BRAF | F- TCAACCACAGGTTTGTCTGC  R- TCACTCGAGTCCCGTCTACC | 58 |
| Bax | F- TTCATCCAGGATCGAGCAG  R- TGAGACACTCGCTCAGCTTC | 56 |
| BcL2 | F- GCCACCTGTGGTCCACCT  R- CTGAAGAGCTCCTCCACCAC | 59 |
| Caspase-3 | F-GCTATTGTAGGCGGTTGT  R-TGTTTCCCTGAGGTTTGC | 60 |
| Caspase -9 | F-TGGAGACTCGAGGGAGTCAG  R-TCGACAACTTTGCTGCTTGC | 59 |
| Caspase -12 | F-AACATGCCTGGCCTCAACAT  R-TCTCACATCCCCAAAAGGTCA | 58 |
| GAPDH | F- AATCCCATCACCATCTTCCA  R- TGGACTCCACGACGTACTCA | 56 |
